# Expansions to the MGDrivE suite for simulating the efficacy of novel gene-drive constructs in the control of mosquito-borne diseases

**DOI:** 10.1186/s13104-023-06533-6

**Published:** 2023-10-05

**Authors:** Jared B. Bennett, Sean L. Wu, Pratima R. Chennuri, Kevin M. Myles, Martial L. Ndeffo-Mbah

**Affiliations:** 1 Mobius Logic, Tysons, VA 22102 USA; 2 Institute for Health Metrics and Evaluation, University of Washington, Seattle, WA 98121 USA; 3 Department of Entomology, Texas A & M University, College Station, TX 77843 USA; 4 Future Fields, Edmonton, AB T5H 0L5 Canada; 5 Department of Integrative Biosciences, Texas A&M University, College Station, TX 77843 USA

**Keywords:** MGDrivE, MGDrivE 2, Gene Drive, Small molecule, Self-eliminating mechanism

## Abstract

**Objective:**

The MGDrivE (MGDrivE 1 and MGDrivE 2) modeling framework provides a flexible and expansive environment for testing the efficacy of novel gene-drive constructs for the control of mosquito-borne diseases. However, the existing model framework did not previously support several features necessary to simulate some types of intervention strategies. Namely, current MGDrivE versions do not permit modeling of small molecule inducible systems for controlling gene expression in gene drive designs or the inheritance patterns of self-eliminating gene drive mechanisms.

**Results:**

Here, we demonstrate a new MGDrivE 2 module that permits the simulation of gene drive strategies incorporating small molecule-inducible systems and self-eliminating gene drive mechanisms. Additionally, we also implemented novel sparsity-aware sampling algorithms for improved computational efficiency in MGDrivE 2 and supplied an analysis and plotting function applicable to the outputs of MGDrivE 1 and MGDrivE 2.

## Introduction

Mosquito-borne diseases, such as malaria, dengue, and Zika, remain a significant public health burden throughout the world [1, 2]. Traditional control methods are expensive and difficult to maintain over long periods of time [2, 3]. This has increased interest in novel vector control strategies, specifically gene drive systems for managing vector-borne disease.

CRISPR-based gene drives (GDs) offer a potentially sustainable approach to vector control via biasing the inheritance of a desired gene in a target population [4–6]. A successful gene drive spreads through an entire population, the desired outcome [7, 8]. GD designs generally follow one of two approaches: population suppression [9], or population replacement [7, 8, 10].

As GD designs become more complex and field trials for wide-scale application are discussed, understanding and predicting how these designs behave under real-world conditions is of increasing importance [11]. Such models need to be flexible enough to accommodate a growing list of innovative new gene drive designs, as well as account for vector movement between populations and seasonal fluctuations in temperature and rainfall [12]. Additionally, models need to reflect epidemiological outcomes, as the intended effects of gene drives are not just entomological, but also reductions in disease incidence [11, 12]. Models incorporating both gene drive and epidemiological dynamics are the foundation of field-trial readiness [12].

The MGDrivE software suite (MGDrivE 1 and MGDrivE 2) represent the state-of-the-art in gene drive modeling. MGDrivE 1 [13] was unique as the first software package capable of simulating diverse, user-specified inheritance schemes and spatially varied landscapes with overlapping-generation population dynamics. MGDrivE 2 [14] builds on the strengths of MGDrivE 1 by incorporating several fundamental improvements. For example, parameters such as variable seasonal dynamics, and pathogen transmission cycles involving mosquitoes and humans to explore epidemiological outcomes, implemented as stochastic Petri nets for efficient and flexible model formulation. The decoupled architecture between model specification and simulation allows efficient storage of models and combinatorial use of fast simulation algorithms from other fields of study.

**Fig. 1:**
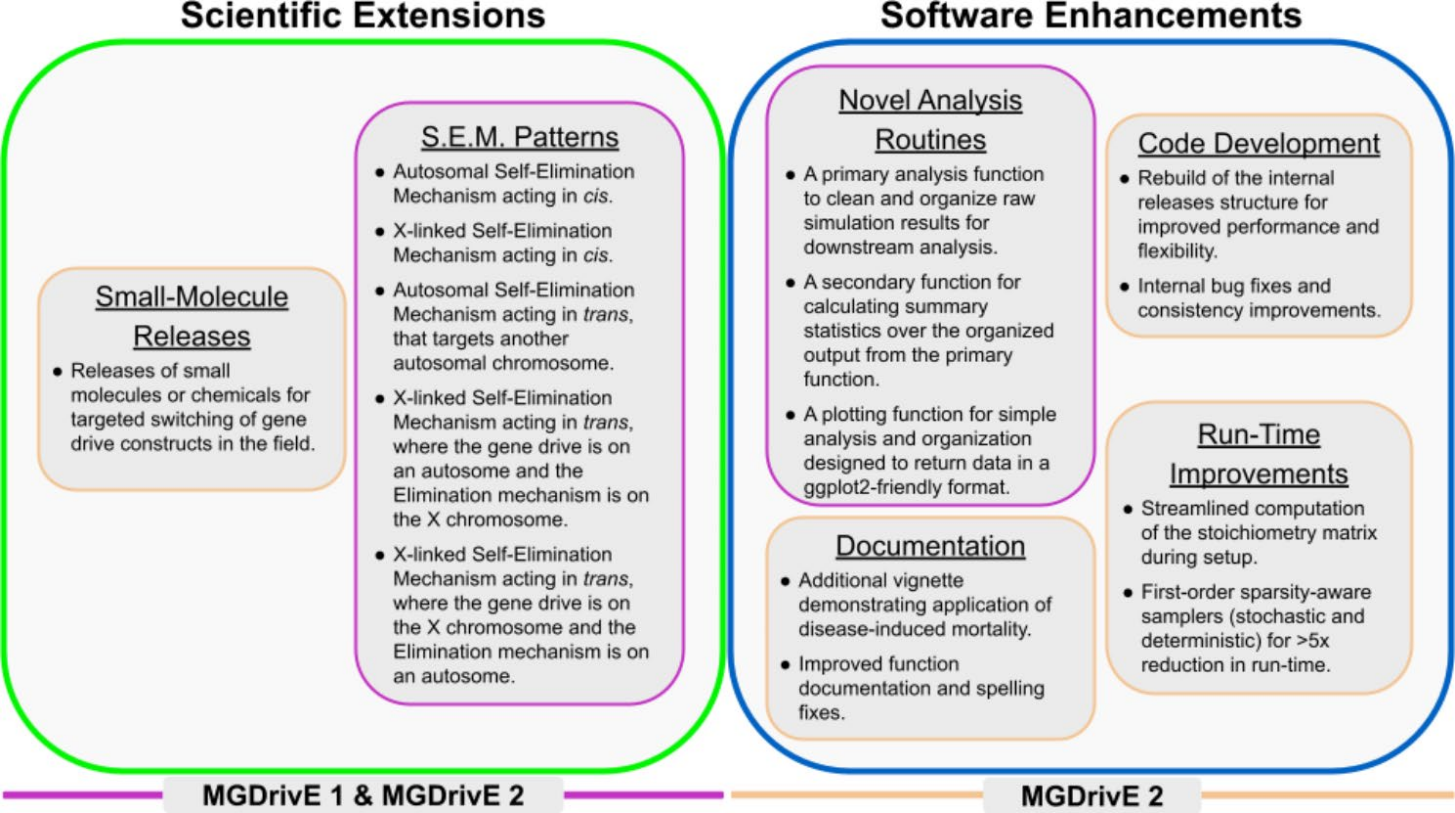
Summary of Extensions and Their Application. Additions to the MGDrivE suite can be organized in 2 ways: Scientific Extensions (green) versus Software Enhancements (blue) or application to version 1 and 2 (purple) versus application to version 2 only (peach). For example, the “Inheritance Patterns” are green and purple, as they represent scientific additions available for MGDrivE 1 and MGDrivE 2. In contrast, the “Documentation” improvements are blue and peach, as they update the MGDrivE 2 software

In this paper, we build on the existing strengths of the MGDrivE 2 software suite by developing additional functionality to simulate new gene-drive constructs and transgenic tools for mosquito-borne disease control. More specifically, additional capabilities for modeling the performance of five novel gene drive designs are included, as well as improved analysis and plotting routines applicable to both the MGDrivE 1 and 2 software. Finally, we upgraded internal functionality for improved computational performance and software maintenance (Fig. 1).

## Main text

### Expanding the MGDrivE 2 repertoire for biodynamic simulation

#### Small molecule-inducible systems

In order to support the development of new gene drive configurations, we added the ability to model strategies incorporating small-molecule-inducible systems [15]. High-resolution temporal or spatial control of gene drives relies on inducible constructs (genetic regulators whose expression can be chemically modulated), and the application of a small molecule for activation or deactivation. To model autonomous control of gene drives with small-molecule activation, we provide 5 self-elimination-based designs and establish the possibility of temporal deactivation [16].

#### Small-molecule activation of gene drive behaviors

Previous designs, such as split gene drive [8] or Daisy-chain gene drive [17], are designed for spatial control. However, they do not provide temporal control over GD activation or deactivation. High-resolution spatiotemporal control is theoretically possible through Inducible Gene Drives, where the GD activity is induced by a small molecule [15]. Super-Mendelian inheritance occurs only after application of a small molecule that influences the expression or stability of a drive component, typically Cas9. Reliance on the external application of a chemical for GD activity provides both spatial and temporal control.

#### Self-elimination mechanism

**Fig. 2:**
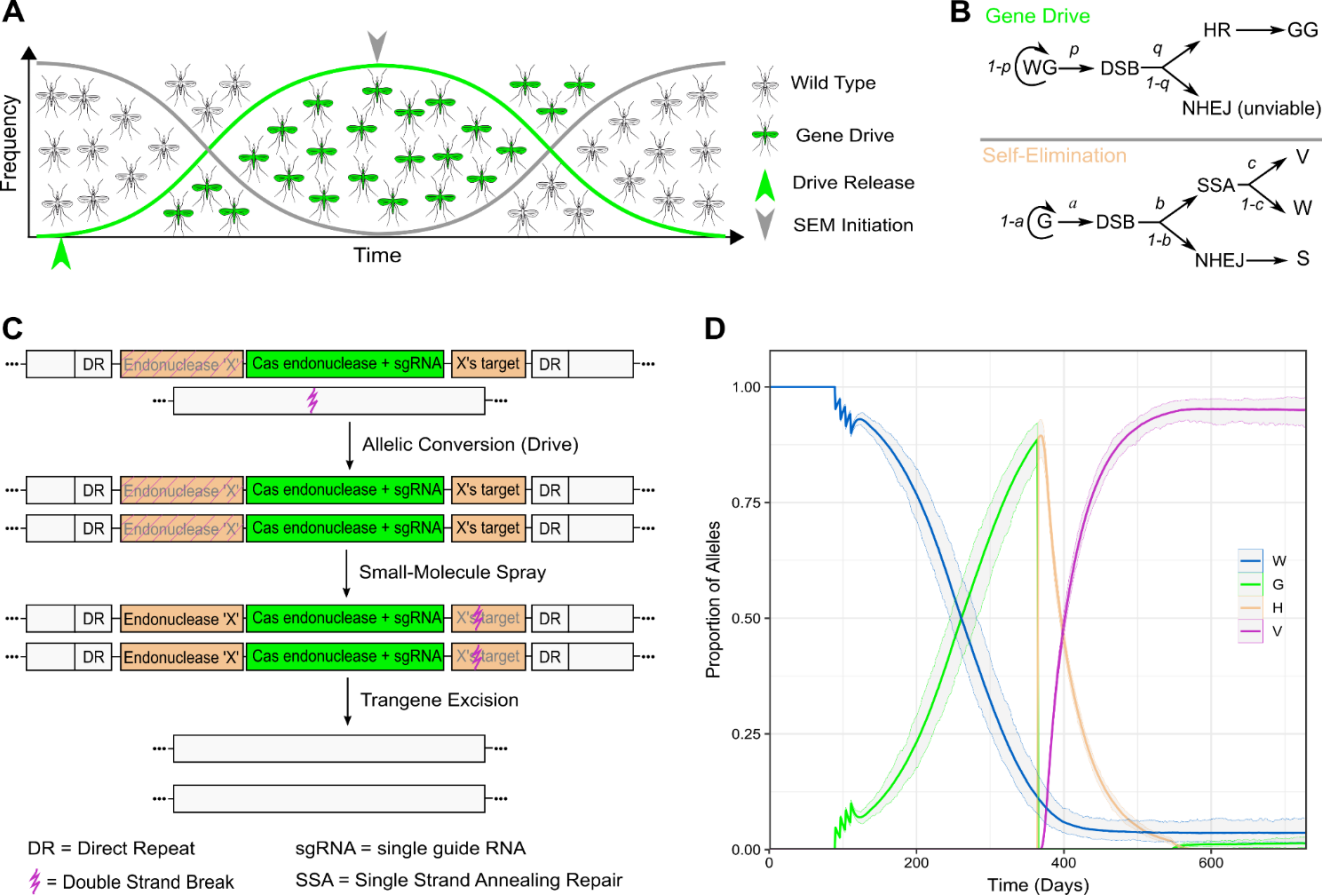
Prototypical Design and Behavior of a *Cis*-acting Self-Elimination Mechanism. **A)** Expected behavior of a GD demonstrating rapid spread followed by subsequent removal and restoration of the wild-type phenotypes. **B)** Simplified mathematical mechanism underlying the SEM design, in the absence of resistance mechanisms. The top panel (“Gene Drive”) depicts the initial behavior of the drive, which cleaves with probability *p* (remaining wild-type with probability *1-p*). Homologous recombination (HR) occurs with probability *q*, leading to a GD homozygote (“GG”), or with probability *1-q* non-homologous end-joining occurs (NHEJ), leading to an unviable genotype. Application of a small-molecule initiates drive (“G”) cleavage (lower panel, “Self-Excision”), with probability *a*, which may be resolved by single strand annealing (SSA) (with probability *b*) or by NHEJ (probability *1-b*). SSA may result in cut-resistant wild-type alleles (“V”) with probability *c*, or susceptible wild-type alleles with probability *1-c* (“W”). NHEJ also may result in an SEM-resistant allele, “S”. **C)** Hypothetical biological arrangement and mechanism of a small molecule activated SEM. The GD transgene (green) is linked to another endonuclease (“Endonuclease ‘X’”) and the associated target site (“X’s target”), flanked by direct repeats (“DR”). Under normal conditions, only the GD is active, and the entire construct propagates through allelic conversion. However, application of a control molecule activates Endonuclease ‘X’, cleaving X’s target site and initiating repair via single strand annealing repair pathway, facilitated by the DRs. The intended outcome is fixation of wild-type alleles that are resistant to drive-mediated cleavage. **D)** Simulated behavior of a simple small molecule activated SEM construct. A single, panmictic population of 2000 mosquitoes was simulated for ~ 2 years. Mosquitoes followed a simple *cis*-acting SEM pattern, where “W” is a wild-type allele, “G” is an active gene drive, “H” is an active SEM, and “V” is the excised cut-resistant wild-type allele that remains. At ~ 3 months of simulation time, we perform 4 weekly releases of 100 SEM-homozygous male mosquitoes. The drive has a cleavage rate of 50% and an HR rate of 100%, generating no resistance alleles. At the 1-year mark, daily small-molecule spraying begins, continuing for ~ 6 months. The activated SEM cleaves at a rate of 50% and repairs alleles 100% of the time, again generating no resistance alleles. Average allele proportions are plotted with 95% empirical quantiles

While several systems have been proposed, and in some cases tested, for controlling gene drives, these strategies rarely address environmental persistence of transgenes [18, 19]. A biodegradable GD is an autonomous homing element that is eliminated from the population after achieving a predetermined successful outcome. Proposed self-eliminating systems consist of a CRISPR-based autonomous homing GD (e.g., Cas9 and sgRNA) engineered to express one or more additional endonucleases with target sites present in the transgene [16, 20]. Cutting by a highly site-specific endonuclease between direct repeats, also engineered into the GD, results in transgene excision by single-strand annealing, and formation of cut-resistant forms of wild-type alleles (V) (Fig. 2). After initially spreading through a population, the cut-resistant wild-type alleles will begin to accumulate, eventually replacing the GD population (Fig. 2). In theory, a biodegradable GD could also be configured so that the self-eliminating mechanism (SEM) could be activated with an inducible system, permitting spatiotemporal control (Fig. 2).

The MGDrivE suite supports exploration of biodegradable GDs through the addition of 5 distinct SEM designs: a *cis*-acting autosomal construct, a *cis*-acting X-linked construct, a *trans*-acting autosomal construct targeting another autosome, a *trans*-acting autosomal construct targeting an X-linked construct, and a *trans*-acting X-linked construct targeting an autosome (Fig. 1). All designs come in three flavors for the gene drive mechanism; a complete version with 2 resistance alleles to simulate in-frame and out-of-frame mutations, a reduced version with only 1 resistance allele, and a simplified version for rapid simulations that has no resistance alleles. For the SEM mechanism, since the target site can either remain intact or be disrupted via NHEJ, the allele “S” can be included to simulate resistance to SEM. We demonstrate an inducible, *cis-*acting autosomal SEM with no resistance alleles, and an intact SEM target site for simplicity and speed (Fig. 2).

### Analysis and performance advancements

Modeling software to support complex simulations must be ergonomic and streamlined even as new features are added. Therefore, we improved MGDrivE 2 in three ways: Analysis and visualization, run-time reductions, and documentation improvements for greater clarity. The MGDrivE suite includes basic analysis routines, which we have augmented with additional analyses and ggplot2 [21] compatibility. Novel sampling algorithms significantly reduce the computational burden of continuous-time simulations. Finally, improved documentation reduces the barriers for using the software and refactored internal implementations make it more amenable to future expansions.

#### Internal analysis functionality for ggplot2 visualization

The MGDrivE suite contains basic analysis and plotting functionality that allows researchers to rapidly develop and investigate small simulations. However, this quickly becomes infeasible for larger, arguably more relevant, simulations. For these simulations, MGDrivE includes basic functions for organizing data, summarizing the complex state of the simulation output (possibly involving state, genotype, or infection status), and calculating simple statistics. Higher-level analysis and complex visualization functionality is provided by the excellent Python package MoNeT-MGDrivE [22], a gene drive targeted extension of MoNeT [23]. MoNeT-MGDrivE contains advanced analysis routines for complex or in-depth analysis, including sensitivity analyses, regression, variable interaction measures, aggregate metrics such as the Window-of-Protection and Time-to-Introgression [24], as well as spatial heterogeneity measures. While this is a powerful tool, exceptional for large-scale studies, it requires a researcher to have experience in a second programming language and software development environment, increasing the complexity and making use more difficult [25].

We simplify the plotting and analysis process for bench researchers by implementing basic MoNeT-MGDrivE functionality within the MGDrivE suite, making tools a researcher needs readily accessible. New functions take the same analyzed output as MoNeT-MGDrivE but output files in a ggplot2 [21] friendly format. Analysis functionality includes sex-specific analysis, flexibility in specifying alleles or genotypes of interest, and automatic calculation of several elemental statistics (max, min, mean, and 50% and 95% quantiles) over user-defined alleles or genotypes of interest.

#### Implementation of first-order sparsity-aware sampling algorithms

MGDrivE 2 represents models as stochastic Petri nets (SPN), separating the model specification from the simulation routines [14]. This separation promotes use and development of fast simulation algorithms from other fields [26]. However, as these representations are equivalent to continuous-time Markov processes, repeated simulation is still computationally demanding. Towards this end, novel routines leverage sparsity within the system to reduce the computational overhead of simulations.

Stochastic simulations in MGDrivE 2 are simulated as competing Poisson processes, one for each transition in the system. Because models are described using SPN for mathematical specification, the intensity of a transition must have at least as many tokens in its input states as the weight of the input arcs. For example, the mortality transition for adult mosquitoes requires at least one adult mosquito in its input state for the intensity to be zero, or, in the language of SPN, for that transition to be enabled. Checking if all input states have at least the required number of tokens can be time consuming during large simulations. Therefore, we implemented first-order sparsity-aware samplers which only need to check that the first input state has a non-zero number of tokens. If the first input state has zero tokens, computation of the intensity can be skipped entirely, because all input states must have at least the minimum number of tokens to be enabled. This enhancement was applied to all samplers supplied with MGDrivE 2 and achieved ~ 5x runtime improvement when generating Fig. 2.

#### Lesser internal improvements and documentation expansion

Developing extensions for MGDrivE 2 required a deep understanding of the original model and its implementation. To reduce the burden of future extensions, the software documentation was improved in three ways:

- New Documentation and Examples
  - All novel inheritance designs come with significant documentation, explaining how each design works, links to relevant publications, and describing how parameters impact inheritance. Additionally, we provide a new vignette with simple examples of disease-induced mortality and a self-elimination mechanism.
- Improvement of Existing Documentation
  - Existing documentation was augmented with additional examples, data structure clarifications for function input or return, spelling corrections, and links between functions of similar functionality or purpose.
- Documentation of Internal Upgrades
  - Significant internal improvements were made to support stability and maintenance of the codebase. Parameter checks were standardized, alleviating errors encountered near extreme ranges of parameter values, code duplication was substantially reduced, and several internal structures were simplified, reducing the computational complexity and maintenance efforts.

## Conclusion

We extend the excellent MGDRivE 2 software packages with the addition of novel scientific functionality, 5 new inheritance patterns based on a recent biodegradable design, and significant computational and software stability improvements. The addition of small-molecule sprays expands the utility of MGDrivE 2 and supports the use of inducible systems. Analysis, visualization, and computational improvements allow researchers to rapidly iterate on designs and parameterization, evaluating innovative ideas for their efficacy prior to expensive cage or field trials. Finally, internal improvements simplify upgrades and additions to the software suite, laying the foundation for future endeavors.

### Limitations

While we provide significant enhancements of the MGDrivE 2 software suite, there are still limitations inherent to our work:

- Use of MGDrivE 2 as a foundation inherits any limitations thereof, such as computational limitations of the R language [27].
- Inheritance patterns are faithful mechanistic representations of our current understanding, which may change with experience, species, or biological experimentation.
- Sparsity-aware samplers rely on sparsity and provide no benefit in densely populated simulations. Additionally, they only apply to the SPN framework of MGDRivE 2 and not to the decoupled samplers.
- For compatibility with MGDrivE 1, analysis functions only work on entomological outputs and not epidemiological ones. For more complex analysis, we recommend using the MoNeT-MGDrivE library.

## Data Availability

Upgraded software package and scripts to generate Fig. 2 are available at https://github.com/mln27/MGDrivE_Disease-Induced-Mortality.

## List of abbreviations

GD: Gene Drive
SEM: Self-Eliminating Mechanism
SPN: Stochastic Petri Net
HR: Homologous Recombination
NHEJ: Non-Homologous End-Joining
SSA: Single-Strand Annealing
